# Agency Modulation of Hippocampal Activity During Spatial Navigation

**DOI:** 10.21203/rs.3.rs-6493656/v1

**Published:** 2025-05-26

**Authors:** Yi-Chuang Lin, Ya-Ting Chang, Charlotte Maschke, Wan-Rue Lin, Yu-Shiang Su, Joshua O. S. Goh

**Affiliations:** 1 Graduate Institute of Brain and Mind Sciences, National Taiwan University; Taipei, Taiwan.; 2 Technische Universität Dresden; Dresden, Germany.; 3 Department of Psychology, National Taiwan University; Taipei, Taiwan.; 4 Neurobiology and Cognitive Science Center, National Taiwan University; Taipei, Taiwan.; 5 Center for Artificial Intelligence and Advanced Robotics, National Taiwan University; Taipei, Taiwan.

## Abstract

The role of navigational agency during hippocampal cognitive map formation remains unclear. To examine this, we measured functional brain activity as participants learned landmarks and paths in virtual mazes either through agentic self-generated movements or guided video tours. Anterior hippocampal responses were generally lower at *objective* nodes (landmarks and junctions) than non-nodes (traversals). Agency lowered hippocampal traversal activity and subsequently resulted in faster and more successful retrieval navigation behavior. Moreover, agency decreased hippocampal functional autocorrelations suggesting lowered responses stemmed from sparse encoding. Finally, hippocampal node encoding corresponded to the extent frontal and temporal responses dissociated nodes from non-nodes. These findings show that agentic spatial navigation affords *subjective* movement decisions during spatial traversal that drive hippocampal sparse encoding of plain space into structured node maps.

## Summary

To code spatial environments, hippocampal (HC) cells ^1,2^ engage selective and topological activities at different places, which constitute cognitive maps ^3–6^. However, while HC activity is modulated by navigational repetition and motivation ^7–10^, how these factors drive HC cognitive map formation remains difficult to validate in the non-human animal studies above. Here, we show that agency in human navigation in humans induces sparse HC activity during map-learning and enhances retrieval navigation. Functional magnetic resonance imaging during agentic spatial navigation detected that the formation of map node states was coded by lower, sparser anterior HC ensemble responses, particularly when traversing between landmarks. Higher activity reflected poorer map encoding instead. Moreover, agency increased frontal and temporal node vs. non-node state dissociations that corresponded with lower HC responses. Our findings link repetitive map exposures and goal processing by demonstrating that *subjective* agentic movement processes in cortical regions drive hippocampal sparse encoding of more accurate cognitive maps. Amongst other inquiries, this framework lays groundwork for examining the role of volition in technology use (e.g. GPS, passive content viewing) on human brain development and aging. Also, a definition of agency in intelligent systems artificial or otherwise might also be developed.

## Main Text:

In rodents ^1,2,11–13^ and humans ^14–17^, spatial environments are represented as cognitive maps instantiated in hippocampal (HC) activity ^13,18^. Specifically, selective and topological firing of HC cell ensembles encode different spatial locations ^3–6^, synonymous with the node states and state associations defining cognitive maps ^19,20^. Nevertheless, how HC cognitive maps are formed to begin with remains an open question. One clue is the modulation of rodent HC place cell responses by rewards, task goals, and motivation ^7–9^. Such findings commonly implicate agency-related processing but are difficult to validate in non-human animals. Here, we applied a functional magnetic resonance imaging (fMRI) experiment to compare HC map-learning responses and retrieval navigation in humans given navigational agency or fixed tours in virtual spaces.

We note that when rodents traverse novel paths interspersed with objects, individual HC cells at first fire to multiple object positions along the path, resulting in high net activity levels in the ensemble with relatively indiscriminate spatial coding ^10^. After repeated traversals, individual cells become selectively active to specific object positions and ensemble activity lowers, i.e., HC spatial coding becomes sparse. Thus, lower and sparse HC activity denotes more selective spatial node states suggestive of cognitive map formation whereas high HC activity levels indicate absence of an effective cognitive map. Rodent path learning, however, affords limited means to distinguish agentic formation of cognitive maps from perceptual desensitization effects in HC sparsification.

**Fig. 1.**
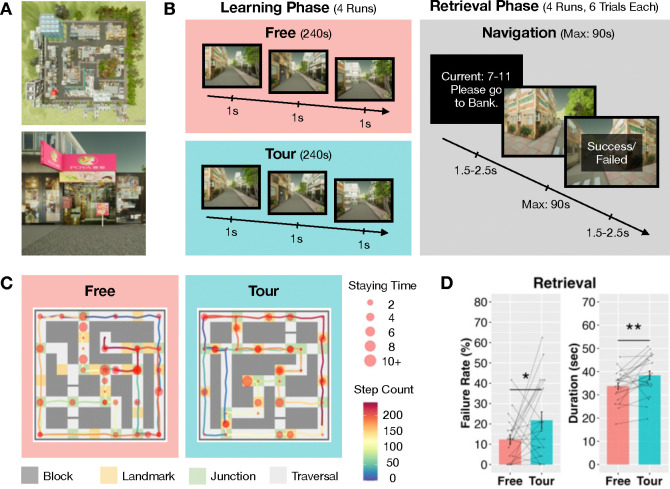
Navigational agency enhanced spatial maze acquisition. **A.** Twenty-one participants navigated a virtual environment resembling street views of local cities. Top: a bird’s-eye-view of the environment (not shown to participants). Bottom: example actual participant first-person view. **B.** Brief schematic of the learning and retrieval phases of the fMRI experiment. Participants learned two different environments under Free or Tour conditions, respectively (see Materials and Methods). Following learning, participants were tested in retrieval trials, where they navigated from various start landmarks to targets within 90 s (see also Supplementary Fig. 1). **C.** Example paths for one participant during Free and Tour map-learning with more steps through landmarks in the latter. **D.** Retrieval navigation performances showed participants had lower failure rates (left) and shorter durations (right) in reaching target landmarks for the Free than the Tour condition. Gray dots and lines denote individual participant performances across conditions. * and ** denote *p*(unc.) < 0.05 and 0.01, respectively, for one-tailed paired t-tests; error bars show standard errors. fMRI: Functional Magnetic Resonance Imaging.

To examine this, we recruited 21 human participants to learn virtual mazes via self-generated (Free) or externally directed (Tour) navigational movements under functional magnetic resonance imaging (fMRI) scanning (Fig. 1A–C) and later perform retrieval navigation from start to target locations. We hypothesized that agentic processing in self-generated movement decisions under navigational agency should provide endogenous discriminative brain signals to the hippocampus to establish spatial node states of more accurate cognitive maps. By contrast, directed tours reduce the role of agency in HC spatial encoding and should yield poorer map knowledge. Correspondingly, we expected that fMRI signals from HC voxels that reflect sparse ensemble activity should be lower during Free than Tour conditions. HC activity should also be lower for node relative to non-node states, such as at *objectively* salient (i.e., junctions and landmarks) relative to non-salient (i.e., traversal path) positions, or when locations are more *subjectively* salient due to agency (i.e., traversal positions from where distal but visible landmarks can be integrated into movement decisions).

In addition, global large-scale representations implicate anterior (aHC) more than posterior (pHC) HC regions ^21–23^. Thus, we expect the effect of agency in modulating environmental cognitive map processing to be greater in aHC than in pHC. Moreover, because sparse spatial coding implies more selective ensemble states, navigational agency should induce finer representational granularity with more distinctive HC responses across different time points and between different neurons ^23,24^. Crucially, agency in map-learning associated with sparse HC responses should improve retrieval navigation success ^25,26^ supporting cognitive map processing rather than merely low signal levels that are inconsequential or even detrimental to navigation. Finally, maze movement involves comparative generation and selection of navigation actions and evaluation of expected and experienced visuospatial sensations post-action. Thus, compared to directed touring, we expected navigational agency to drive engagement of decision processing areas, including the supplementary motor, medial and lateral frontal, and striatal regions ^27–38^. Moreover, greater engagement in these regions to dissociate node and non-node states should correlate with sparser HC map-learning responses.

## Results

### Agency reduced anterior hippocampal responses

**Fig. 2.**
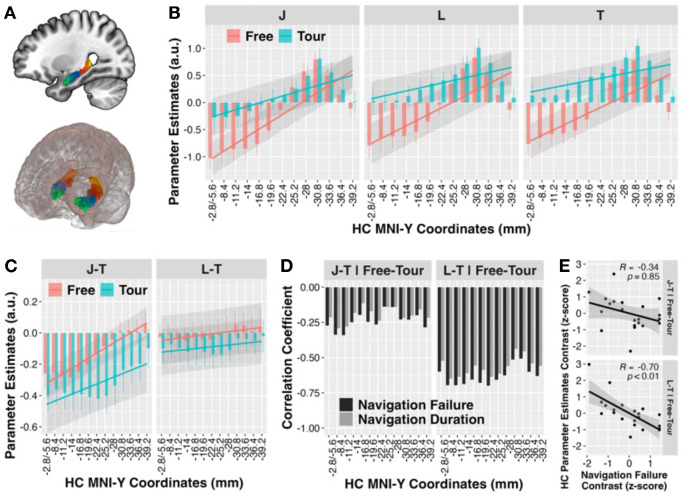
Navigational agency induced lower HC responses and enhanced behavioral performances. **A.** 3D plots showing the left and right *a priori* anatomical HC ROIs derived from the AAL atlas, separated into 13 coronal sections marked with different colors. **B.** HC maze learning responses in each coronal section over the anterior to posterior gradient for J, L, and T location states during Free (pink) and Tour (cyan) conditions. Mixed-model predictions of gradient trendlines are overlaid with shaded 95% confidence intervals. **C.** J-T and L-T response contrasts along the HC gradient. **D.** Pearson correlation coefficients between agency-modulated HC neural response contrasts [J - T]_Free-Tour_ and [L - T]_Free-Tour_ with the corresponding navigation behavioral contrasts along the anterior to posterior HC. **E.** Scatter plots illustrating the highest correlations between Free - Tour navigational failure contrast and [J - T]_Free-Tour_ (top) and [L - T]_Free-Tour_ (bottom) response contrast in the HC sub-ROI y = −11.2 mm (top) and −22.4 mm (bottom), respectively. Black dots denote each participant. HC: Hippocampal, ROI: Regions-of-Interest, AAL: Automated Anatomical Labels, J: Junction, L: Landmark, T: Traversal, FDR: False Discovery Rate.

Neural responses during each navigation condition (Free, Tour) were estimated for junctions (J), landmarks (L), and traversal (T) states in *a priori* combined bilateral HC regions-of-interest (ROI) over an anatomical gradient of 13 anterior to posterior coronal sections (y-coordinate positions; see Materials and Methods) (Fig. 2A, B). ANOVA of these HC responses (Supplementary Table 1) revealed significant main effects of navigation condition (*F*_1,1606_ = 14.3, *p* < 0.001), maze position state (*F*_2,1606_ = 8.26, *p* < 0.001), and HC gradient (*F*_1,1606_ = 43.8, *p* < 0.001), with interactions between condition × gradient (*F*_1,1606_ = 24.8, *p* < 0.001), and state × gradient (*F*_2,1606_ = 3.89, *p* = 0.021). The three-way condition × state × gradient interaction was not significant. As expected, planned contrasts of the linear model coefficients (Supplementary Table 2) showed that overall HC activity was lower during Free than Tour conditions (*β*_Tour-Free_(S.E.) = 0.901 (0.238), t_1606_ = 3.78, p < 0.001), lower for junction than traversal states (*β*_J-T_(S.E.) = −0.323 (0.086), *t*_1606_ = −3.77, *p* < 0.001), lower in anterior than posterior HC ROIs (*β*_Gradient_(S.E.) = 0.103 (0.016), t_1606_ = 6.62, p < 0.001), with the gradient effect steeper for Free than Tour conditions (*β*_Gradient.(Tour-Free)_(S.E.) = −0.060 (0.012), t_1606_ = −4.98, p < 0.001) and for junctions than for traversals (*β*_Gradient.(J-T)_(S.E.) = 0.032 (0.012), t_1606_ = 2.65, p = 0.008). State response differences did not significantly differ across navigation conditions in this model.

Importantly, we applied a linear model of within-subject HC response contrasts for junction and landmark responses, separately, against the traversal responses of the respective Free and Tour conditions to adjust for possible baseline differences in state response estimates across condition sessions (Fig. 2C, Supplementary Table 3, 4). Planned comparisons showed lower responses to junctions than traversals (*β*_J-T_(S.E.) = −0.323 (0.059), *t*_522_ = −5.48, *p* < 0.001) with this response difference being greater in size in anterior than posterior HC ROIs (*β*_Gradient.(J-T)_(S.E.) = 0.032 (0.006), *t*_522_ = 5.44, *p* < 0.001). Critically, the response contrast was less negative during Free than Tour conditions (*β*_(J-T).(Tour-Free)_(S.E.) = −0.134 (0.060), *t*_522_ = −2.26, *p* = 0.024), consistent with reduced traversal responses under navigational agency, as expected. While there were also trends for lower responses to landmarks than traversals that were also reduced during the Free relative to the Tour condition, these did not reach significance.

### Agency increased hippocampal response granularity

**Fig. 3.**
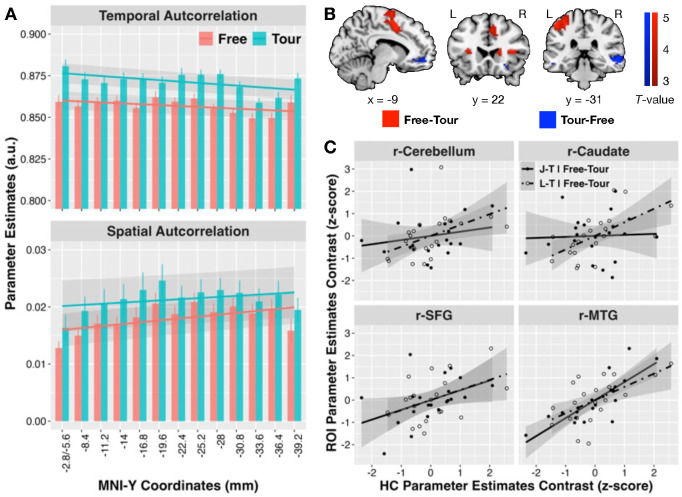
Navigational agency increased HC granularity, and decision-making circuits supported HC in encoding subjective nodes during space acquisition. **A.** HC temporal autocorrelation (top panel) and spatial autocorrelation (bottom panel) along the anterior-posterior axis under the Free (pink) and Tour (cyan) conditions. Mixed-effects model prediction lines are overlaid on top of the HC responses, with the shaded regions marking 95% confidence. **B.** Whole-brain activation under the Free-Tour (red) and Tour-Free (blue) contrasts. Contrasts were significant at whole-brain cluster-wise *p*(FDR) < 0.05. See Table 3 for the full list of contrast peaks. **C.** Scatter plots showing associations between neural contrasts between bilateral HC ROI and representative whole-brain functional contrast ROIs (see Table 1) including left medial OFC (MNI coordinate: −9, 50, −7), right caudate (19, 22, 11), right SMA (28, 14, 62), and right MTG (61, −31, −7). Each dot denotes z-scored data from one participant for the given contrast. Regression prediction lines are overlaid on scatter plots, with shaded regions marking 95% confidence. HC: Hippocampal, FDR: False Discovery Rate, ROI: Regions-of-Interest, AAL: Automated Anatomical Labels, MNI: Montreal Neurological Institute, OFC: Orbitofrontal Cortex, SMA: Supplementary Motor Area, MTG: Middle Temporal Gyrus.

To validate lower HC responses as reflecting selective and sparse encoding, we examined the *granularity* of HC activity ^23,24^ during map-learning navigation based on how *low* temporal (voxel-response-pattern correlations over time) and spatial (response-time-series correlations over voxels) autocorrelations were in the HC ROIs (see Materials and Methods). HC activity generally showed high temporal autocorrelation but low spatial autocorrelation values (Fig. 3A). Critically, HC activity temporal (*F*_1, 522_ = 182, *p* < 0.001) and spatial (*F*_1, 522_ = 44.1, *p* < 0.001) autocorrelations were significantly lower for Free than Tour conditions (Supplementary Table 5, 6), consistent with higher functional granularity or sparsity under navigational agency. Interestingly, temporal autocorrelations decreased from anterior to posterior HC regions (*β*_Gradient_(S.E.) = −5e-4 (2e-4), t_522_ = −2.62, p = 0.009) and increased for spatial autocorrelations (*β*_Gradient_(S.E.) = 3e-4 (1e-4), t_522_ = 3.44, p < 0.001), albeit there were no interactions with navigation condition.

### Hippocampal response sparsity enhanced retrieval

During map retrieval, participants had lower failure rates (t_20_ = −2.05, *p* = 0.027) and shorter durations (t_20_ = −2.78, *p* = 0.005) finding target landmarks within the 90s limit for mazes learned under Free than Tour conditions (Fig. 1D). This was despite participants passing by fewer than the total 12 landmarks during the Free condition (mean (S.E.) landmarks per run per participant: 8.18 (0.39); t_20_ = −9.69, *p* < 0.001) and lower overall HC responses for Free than Tour map-learning conditions as above. Importantly, participants in which HC traversal responses were lower during Free than Tour map-learning, indexed by reduced neural contrast between landmark and traversal responses, correspondingly showed greater reductions in map retrieval navigation failures (r_19_ range: −0.51 to −0.70, all *p*’s < .01) and durations (r_19_ range: −0.44 to −0.65, all *p*’s < .05) in Free than Tour conditions (Fig. 2D–E). HC map-learning junction and traversal contrasts were also negatively correlated with retrieval navigation failures and durations, albeit these trends did not reach significance.

### Whole-brain responses and hippocampal node coding

Whole-brain voxel-wise contrasts of map-learning responses revealed greater distinction of objective spatial nodes during Free than Tour conditions across non-HC regions identified using J-T contrasts in the right cerebellum and fusiform gyrus, and L-T contrasts in the right middle and superior frontal gyri (Table 1, Fig. 3B). Map-learning responses were also generally higher for Free than Tour conditions across bilateral rolandic operculum, left precentral, and supplementary (SMA) motor, and right caudate, inferior frontal (opercular), and cerebellar areas. By contrast, neural responses were higher for Tour than Free conditions in bilateral middle temporal (MTG), and inferior frontal gyri (orbital), and left medial orbitofrontal cortex (OFC). Consistent with *a priori* HC ROI findings, voxel-wise contrasts identified greater L-T contrast during Free than Tour conditions in the left hippocampus, and higher Tour than Free responses in the right hippocampus, albeit these did not surpass whole-brain multiple comparisons correction.

ROIs were defined from the above 17 non-HC contrast peak loci (Table 1) to examine how navigational agency effects on HC responses (across 13 sections) to objective nodes (J, L) vs. non-node (T) states (Fig. 2C) were associated with the same node vs. non-node dissociations in implicated non-HC systems. As seen in Table 1 rightmost columns, all contrast correlations were positive (representative ROI scatterplots in Fig. 3C). Specifically, L-T contrasts in the hippocampus were less negative during Free than Tour conditions to the degree that they were also greater for Free than Tour conditions across several non-HC ROIs. These encompassed ROIs showing greater J-T or Free-Tour contrasts including right superior, middle, and inferior (opercular) frontal, caudate, rolandic operculum, and cerebellar areas, as well as ROIs showing greater Tour-Free contrast including left medial orbitofrontal, bilateral inferior (orbital) frontal, and middle temporal areas. Similarly, less negative J-T contrasts during Free than Tour conditions in the hippocampus were associated with the same contrast in the right superior, middle, and inferior (opercular) frontal, as well as left medial orbitofrontal, and bilateral middle temporal, and inferior frontal (orbital) ROIs. Overall, greater functional sparsity in the hippocampus during Free than Tour conditions was associated with agency-related enhancement of node vs. non-node dissociations in non-HC ROIs.

## Discussion

We found evidence that formation of spatial cognitive maps ^19,20,39^ in the human hippocampus involves sparsification of ensemble neural activity to node positions, consistent with transition from non-selective to selective hippocampal place responses in rodent path-learning ^10^. Specifically, navigational agency lowered hippocampal ensemble activity related to node processing during map learning that enhanced navigational performance. Moreover, agentic operations across non-HC systems might underlie hippocampal sparse functioning to approach more system-level graph-like understanding of the environment ^25,26^.

Notably, agency-related hippocampal response lowering generalized across junctions and landmarks, as well as traversal position states. Moreover, smaller map-learning response differences between landmark and traversal states predicted more successful and faster goal navigation at retrieval. Thus, cognitive map formation did not simply involve increased hippocampal dissociative encoding of nodes relative to non-nodes *based on objectively defined spatial frames-of-reference*. Rather, we suggest that hippocampal cells encoded traversals as *subjective nodes under agency*, hence the detection of similarly sparse neural ensemble activity between traversal and landmark states. Specifically, in directed tours, movement planning and node state integration were less controllable, resulting in most traversal locations having non-node status. By contrast, navigational agency required movement planning and decision operations as well as active processing of scenes at various locations that afforded online encoding of traversal positions as node states like landmarks or junctions.

Not surprisingly, the non-hippocampal brain areas detected that expressed higher map-learning activity or node contrasts under agency have been implicated in visually guided navigational action decision and control processing ^34,40–44^. These include the caudate, superior, middle, and inferior (opercular) frontal gyri, rolandic operculum, and cerebellum. Moreover, HC contrast associations with these regions were restricted to the right hemisphere. By contrast, directed touring induced higher responses than agency in temporal, inferior frontal (orbital), and medial orbitofrontal regions, implicated in higher-level object recognition and goal processing ^45–47^. HC contrast associations with these were homologous across both hemispheres. Thus, agency and directed touring modulated distinct neural systems despite comparable visuospatial input during maze navigation. We note that the medial orbitofrontal cortex, typically implicated in decision-making, was less active during navigational agency than directed touring in our study. Nevertheless, this is in line with its role in goal-related object processing, particularly when volitional navigational decisions are prohibited, and more internal monitoring of landmarks is needed ^27,29–32^. Together, neural signals from both action decision and object processing systems have complementary associations with HC labeling of node states during cognitive map formation. By extension, agentic processing in these regions might also contribute to offline hippocampal states such as in self-generation of imaginary spaces or routes which we perceptually navigate independently of environmental sensations. Studies manipulating imagined vs. real spatial navigation would shed further light on how neural coding of movement control is integrated with HC spatial encoding sans sensory associations.

Beyond spatial cognitive maps, the node vs. non-node neural state motif ^48^ has been observed for other information domains, e.g., 2-dimensional mapping of bird neck and leg lengths ^49^ or sequences of objects ^50^ reflecting its fundamental operation in the brain. We add to this by highlighting the subjective and dynamic aspects of cognitive maps, with new nodes being autonomously defined or removed over time. Indeed, agentic processes modulated HC responses of traversal positions, for which there is no salient objective definition, to almost the same level as landmarks, interpreted as subjective node encoding. However, although associated with better navigation performance, exhaustive subjective node encoding of cognitive maps of vast spatial environments is not optimal for a finite neural system. Thus, agency-based enhancement of cognitive map formation likely has an upper efficiency limit, and other strategies are needed for our neural systems to maneuver in the effectively infinite environment. In this vein, future studies might examine constraints in the brain for using cognitive maps to represent large spaces or contexts before switching to more “just in time” compiling operations and what neural operations are entailed.

Finally, the effect of agency on cognitive map processing was dissociated along the hippocampal longitudinal gradient. Specifically, the navigation task condition context modulation of node vs. non-node responses was greater in the anterior than posterior hippocampus, consistent with larger functional temporal and spatial representational field sizes in the former ^21–24^ While functional granularity differences over the hippocampal gradient were not large in size, we note that temporal autocorrelations of voxel fMRI patterns were also somewhat stronger in the anterior hippocampus. However, unlike previous findings, spatial fMRI autocorrelations were stronger in the posterior hippocampus. We speculate that HC spatial autocorrelations were affected by the dynamic nature of our open maze navigation protocol, compared to more restricted path navigation designs in previous studies ^24^. Specifically, our fMRI measurements included self-determined transient periods of active maze exploration or pauses for route evaluation or planning. Inter-voxel spatial autocorrelation estimates, which assume homoscedasticity over entire voxel time series, might have been sensitive to the intermittent global processing variations, particularly in anterior hippocampus. By contrast, temporal autocorrelations captured inter-voxel activity pattern differences in HC neurons of a given time point with every other time point and were less sensitive to time series sequential dynamics. Overall, HC functional temporal and spatial autocorrelations were lower in Free than Tour conditions, indexing greater functional granularity in the former and supporting the role of agency in generally increasing HC functional sparsity.

Recent behavioral studies have suggested that reliance on navigational software has undermined human spatial memory abilities ^51,52^. Indeed, participants in our study exhibited poorer navigation retrieval performance for mazes learned under guided tours compared to self-navigation. Our findings highlight a neural mechanism underlying how navigational software usage might lead to spatial memory difficulties via reduced HC communication with action-based decision-making brain regions at non-salient locations, resulting in less accurately represented cognitive maps with fewer spatial nodes. Indeed, our study showcases that measurable neural and behavioral improvements in spatial navigation processing are afforded within the same person when one explores on one’s own. To our knowledge, ours is the first study to examine the effects of spatial navigational agency on human hippocampal functional activity in relation to system-wide brain processing. Beyond hippocampal place cell response adaptation through passive experience with environmental features, agentic movement decision processing in the rest of the brain also regulates place cell activities and alter mental representations of space in a non-trivial manner.

## Methods

### Participants

Twenty-one participants (11 females; 23.7 ± 2.3 years old) underwent two fMRI experiments each. All participants were right-handed, with normal or corrected to normal vision, without neurological or psychological disorders, or counter-indications for MRI scanning. Informed consent approved by National Taiwan University Hospital Research Ethics Committee was obtained in written form from all participants before the experiment.

### Stimuli

Virtual maze environments were generated using Unreal Engine (Version: 4.18.3) (Fig. 1A). Photographed real-life street views in Taipei city were used to construct landmark buildings in two similar mazes occupying 12 × 12 virtual grid areas. Both mazes consisted of 12 landmarks distributed across nine city blocks of different configurations with similar numbers of junctions (13 and 14) and between-goal distances. First-person maze views were then screenshot at each non-landmark location with 45° view rotations and at landmarks with 22.5° rotations. Screenshots were then used to build a discrete first-person navigation maze paradigm delivered using E-prime 2.0 software (Psychology Software Tools, Pittsburgh, PA). The between-trial fixation stimulus was a 1 × 1 cm white cross in the center of a black screen with durations jittered from 1.5 to 2.5 seconds.

Throughout the experiments, participants used right-hand button presses to indicate judgments and engage movements. Index, middle, ring, and pinky finger presses indicated left, forward, right, and backward movements, respectively. Movements were restricted to one grid step per press with one second per step to instantiate discrete and slow navigation and facilitate fMRI signal modeling of neural responses with hemodynamic response function (HRF) convolution (see fMRI analysis below).

### Familiarization Procedure

Before each formal experiment, we familiarized participants with the appearances of the 12 landmarks of each maze using recognition testing with feedback on landmark (food, retail, or service outlets) logo and name pairings. Participants were repeatedly tested on the logo-name pairs until there were no errors. Participants were then familiarized with the navigation controls in a simplified version of the formal experimental maze with one learning and retrieval run, both with a navigation trial with one landmark to memorize and navigate to.

Navigational condition during familiarization maze learning was matched to the formal experimental condition (Free or Tour; Fig. 1). The navigation condition of the second experiment remained undisclosed to participants until after the completion of the first experiment. At the beginning of the familiarization map-learning run, participants read instructions on motion control and the one landmark to memorize. In the Free condition learning phase, participants were placed in the maze and had agency to navigate the maze environment using button presses and memorize landmarks, junctions, and traversal paths. Virtual movement was implemented as one grid step per press with one second per step. At retrieval familiarization, participants were placed at the same start location as in the learning run, made direction and distance judgments to target landmarks, and navigated to the landmarks within a 30-second limit (Supplementary Fig. 1). Retrieval trials were repeated until the participants reported confidence with navigational motion controls. In the Tour condition, participants viewed one pre-recorded video clip tour of the maze that presented stimuli the same way as in the Free condition except that the routes were predetermined to cover the maze and match a representative Free condition navigation path. Free navigation and Tour video clips started from the same maze location, with the one navigation trial in the run taking 30 secs maximum.

### Experimental Procedure

Participants completed the two navigation experiments on two different days with counterbalancing across the two conditions (Free and Tour) and two mazes. On the first day, participants were randomly assigned to either the Free or Tour condition and one of the mazes. Participants returned within one week to complete the other experimental condition in the other maze. On both experiment days, participants underwent the corresponding familiarization session followed by the formal experiment.

Per condition (Free or Tour), the formal experiment was conducted during MRI scanning with four learning runs then followed by four retrieval runs, each including six trials (Fig. 1A, B; Supplementary Fig. 1). For each learning run, participants were tasked to memorize as many of the twelve landmark locations as possible. In the Tour condition, participants watched four different pre-recorded video clips in the same order, where each route was predetermined and traveled through all twelve landmarks. Free navigation and video clips started from the same maze location, with each run taking 240 secs. For each retrieval run trial, participants were first placed at various start locations with text instruction on the target landmark. Participants then made direction and distance judgments, following which they navigated to target landmarks within a 90-second limit.

### Behavioral Analysis

Statistical tests of behavioral data were conducted using R (v4.3.2; R Core Team 2023) (*53, 54*). We sought to validate that subsequent retrieval navigation performance differences between Free and Tour conditions were due to agency rather than differences in visual exposure to the maze during learning. To this end, locations, orientations, and onset time points for each movement within each maze were recorded for each participant. From these, we derived the frequencies that each maze coordinate location was traversed. For both mazes, paired-sample *t*-tests contrasted group-level traversal frequency differences for each location between the Free and Tour conditions. Equal or lower coordinate traversal frequencies for Free relative to Tour conditions indicate that it was unlikely that better spatial navigation retrieval performances, if present, were due to greater visual maze exposure in the former condition.

For retrieval runs, beyond the navigation movement data, direction and distance judgments were additionally recorded, albeit these latter variables were not of central interest and not further considered here (see Supplementary Text, Direction and Distance Judgement). Critically, we compared landmark finding navigation failures for mazes learned under Free vs. Tour conditions. For each trial, retrieval failure was set as 1 when participants failed to reach the target landmark within 90 sec and 0 otherwise. For successful trials, we compared differences in time taken to reach target landmarks under Free vs. Tour learning conditions. Paired-sample *t*-tests were then applied to evaluate the effect of learning conditions on navigation retrieval failures and time taken.

### fMRI Data Acquisition

fMRI data were acquired using a 3T SIEMENS MAGNETOM Skyra scanner with a 32-channel head coil at the Taiwan Mind and Brain Imaging Center, National Cheng-Chi University, Taiwan. For each participant, 38 T2-weighted structural image axial slices parallel to the anterior-posterior commissural plane were collected for coregistration with voxel resolution 1 × 1 × 4 mm, in-plane matrix 256 × 256, field of view (FOV) 256 × 256 mm, repetition time (TR) 7480 ms, echo time (TE) 102 ms, and flip angle (FA) 150°. Functional volumes were collected using an echo-planar imaging (EPI) sequence with 38 axial slices coplanar to the T2 image, voxel resolution 2.8 × 2.8 × 3 mm, matrix 78 × 78, FOV 220 × 220 mm, TR 2400 ms, TE 30 ms, and FA 80°. A slightly higher functional voxel resolution with minimal TR extension was applied in order to facilitate examining spatial differences in responses along the length of the hippocampus. Learning runs consisted of 100 volumes (240 s / 2.4 s TR) each, whereas retrieval runs had variable functional volumes pending participant navigation performance with a cap at 225 volumes (90 s × 6 trials / 2.4 s TR). T1-weighted magnetization prepared rapid gradient echo (MPRAGE) image was collected for spatial normalization with 192 sagittal slices, voxel resolution 1 × 1 × 1 mm, in-plane matrix 256 × 256, FOV 256 × 256 mm, TR 2500 ms, TE 4.37 ms, and FA 7°.

### fMRI Data Preprocessing and Whole-brain Analysis

We focused on fMRI data for maze learning runs in this study on neural correlates underlying spatial map formation. fMRI data preprocessing was conducted using SPM12 ^53^ in MATLAB R2022b (Natick, Massachusetts; The MathWorks Inc., 2022). For each participant’s functional images, we applied head motion and slice-timing correction, co-registration to T1 images via the coplanar T2 images, spatial normalization to the East Asian brain template in Montreal Neurological Institute (MNI) space and smoothing using a 3D 8 mm full width at half maximum (FWHM) Gaussian kernel.

For each participant, the above preprocessed fMRI responses during learning were estimated using subject-level general linear models (GLMs) composed of regressors for the delta onsets of maze location states convolved with the hemodynamic response function (HRF). Location states included junctions (J), landmarks (L), traversals (T), and navigating dummies (D: movement pauses > 5 s). Six motion regressors were included as covariates. Overall, the GLM had 90 regressors (2 conditions, Free/Tour; 4 locations states, J/L/T/D; 6 motion covariates; 4 learning runs) with contrasts averaging estimates across runs to yield individual whole-brain estimates of responses for J, L, and T locations for Free and Tour conditions.

Individual whole-brain estimates were fed into group-level paired t-tests to identify voxels showing spatial node processing that were influenced by navigational agency. We applied contrasts (Free > Tour and Tour > Free) to identify brain areas in which responses differed by agency on average. Importantly, we considered L and J locations as objective spatial nodes and T states as non-nodes and applied the contrasts (J - T)_Free - Tour_ and (L - T)_Free - Tour_ to identify brain areas in which objective node and non-node coding was modulated by agency. Whole-brain significance thresholds were set using voxel contrast *p*(unc.) < 0.001 and cluster sizes at family-wise error (FWE) *p* < 0.05 for detection within a gray matter mask computed with AlphaSim at 1000 iterations per group-level contrast ^54^.

### ROI Definition and Analysis

Per our hypothesis, *a priori* anatomical ROIs of bilateral HC were defined using the Automated Anatomical Labeling (AAL) atlas in MarsBar (https://marsbar-toolbox.github.io/). AAL HC ROIs were divided into 13 sub-ROI sections with similar voxel numbers (M(SD) = 48.31(11.47)) using equal spacing of 2.8 mm along the y-axis in MNI space from −2.8/−5.6 mm to −39.2 mm. The anterior most section included two y-coordinates to account for low HC signals in some participants. The anterior most HC section was coded as 0 in the regression model through to the posterior most section, which was coded as 13. Functional ROIs were also defined from group-level whole-brain contrasts as spheres of 6 mm radius around peak contrast voxels as defined above. Mean ROI response estimates were extracted from each individual’s whole-brain estimates for J, L, and T location states and Free and Tour conditions.

Statistical analyses of extracted ROI responses were conducted using R (v4.3.2; R Core Team 2023) with the *nlme* package for mixed-effects modeling. For all ROI results, significance was based on *p*-values corrected to < 0.05 false discovery rate (FDR). Paired-sample *t*-tests were used to evaluate how ROI mean location state response differences (J/L/T) during maze learning were modulated by navigation agency conditions (Free/Tour). To determine how agency modulated HC activity across different sub-regions, we applied a mixed-effects model on the *a priori* HC sub-ROI data with condition (Free/Tour) and gradient (13 levels from anterior to posterior positions) as interacting fixed effects and participants as a random effect. Pearson’s correlations were used to evaluate associations between specific individual HC sub-ROI and whole-brain functional ROI neural response contrasts during learning (J>T|Free>Tour, L>T|Free>Tour), and retrieval navigation performance contrasts (Navigation Failure|Free>Tour, Navigation Duration|Free>Tour).

Examination of autocorrelation indices of HC responses was based on non-smoothed functional image data (only motion and slice time corrected with anatomical co-registration and spatial normalization). Data were extracted from non-ROI-averaged voxel-level response estimates from the 13 HC sub-ROIs for each participant for each learning run per condition. For temporal autocorrelation, we calculated Pearson’s correlation coefficients between all voxel response patterns at each TR with all other TRs within each run. An exponential temporal weighting of *γ* = 0.1 relative to each TR was applied to coefficients with other TRs to reduce the influence of distal TRs. Weighted correlation coefficients were normalized using the Fisher-*Z* transform and averaged across runs to yield temporal autocorrelation indices for each sub-ROI per participant per condition. Lower values indicated that voxel responses were less similar across TRs reflecting sparser temporal dynamics in ensemble network activity. For spatial autocorrelation, Pearson’s correlation coefficients were calculated between time series of all voxels of each sub-ROI. Coefficients were normalized using the Fisher-Z transform, excluding diagonal identities, and averaged across runs within each sub-ROI per participant per condition. Lower values indicated that responses were less similar between voxels reflecting sparser coherences across space.

## Figures and Tables

**Table 1.** Activation peak details for whole-brain voxel-wise contrasts of the effects of navigational condition (Free, Tour) and maze position state (J: Junction, L: Landmark, T: Traversal).

Whole-brain Contrast	Brain Region	Hemisphere	k	t	x	y	z	Contrast ρ with HC	

								(J - T)_Free-Tour_	(L - T)_Free-Tour_

(J - T)_Free-Tour_^1^	Cerebellum	Rt	542	6.19	19	−48	−19	0.12	0.24
	Fusiform Gyrus	Rt	-	5.28	28	−36	−22	0.18	0.36
	Cerebellum	Rt	-	5.26	16	−59	−19	0.19	0.49*
	
(L - T)_Free-Tour_^2^	Middle Frontal Gyrus	Rt	80	4.78	25	11	47	0.44*	0.44*
	Superior Frontal Gyrus	Rt	-	3.42	28	14	62	0.44*	0.45*
	Hippocampus^5^	Lf	5	3.75	−34	−39	7	-	-
	
Free - Tour^3^	Precentral Gyrus	Lf	713	8.88	−40	−17	59	0.14	0.11
	Supplementary Motor Area	Lf	552	6.05	−3	0	56	0.42	0.22
	Inferior Frontal Gyrus (oper.)	Rt	244	6.01	39	17	11	0.48*	0.47*
	Rolandic Operculum	Rt	-	4.58	50	6	11	0.32	0.57**
	Caudate	Rt	-	4.55	19	22	11	0.04	0.61**
	Cerebellum	Rt	237	7.47	19	−50	−19	0.13	0.24
	Rolandic Operculum	Lf	126	5.14	−45	−3	11	0.14	−0.08
	
Tour - Free^4^	Middle Temporal Gyrus	Rt	319	5.22	61	−31	−7	0.81***	0.59**
	Medial Orbitofrontal Cortex	Lf	152	4.66	−9	50	−7	0.55*	0.46*
	Middle Temporal Gyrus	Lf	95	4.62	−59	−14	−22	0.78***	0.50*
	Inferior Frontal Gyrus (Orb.)	Rt	77	4.73	44	34	−10	0.60**	0.63**
	Inferior Frontal Gyrus (Orb.)	Lf	71	4.45	−42	36	−10	0.48*	0.73***
	Hippocampus^5^	Rt	19	4.42	33	−17	−19	-	-

1 k > 50

2 k > 54

3 k > 52

4 k > 47

5 p-values were < 0.001 uncorrected in these ROI but did not surpass whole-brain cluster-wise p(FWE) < 0.05.

Estimates of J and L response differences with T during Free contrasted with Tour conditions were extracted from ROIs (regions-of-interest) defined around peaks. ROI contrast estimates were correlated with the same contrast in the whole HC ROI (two rightmost columns).

*, **, *** denote p(FDR) < 0.05, 0.01, and 0.001, respectively.

t: Contrast t-statistic; k: cluster size in voxels; x, y, z: Template space coordinates; ρ: Correlation coefficient; HC: Hippocampal; Lf: Left; Rt: Right, FDR: False Discovery Rate.
